# Soft Computing Methods in Civil Engineering

**DOI:** 10.1155/2015/605871

**Published:** 2015-03-25

**Authors:** Siamak Talatahari, Vijay P. Singh, Amir H. Alavi, Fei Kang

**Affiliations:** ^1^Department of Civil Engineering, University of Tabriz, Tabriz, Iran; ^2^Department of Biological and Agricultural Engineering, Texas A&M University, College Station, USA; ^3^Department of Civil and Environmental Engineering, Michigan State University, Engineering Building, East Lansing, MI 48824, USA; ^4^Dalian University of Technology, Dalian, China

Demand for lightweight, efficient, and low cost structures seems mandatory because of growing realization of the rarity of raw materials and rapid depletion of convention energy sources. This requires engineers to be aware of optimization techniques. Designing, analyzing, and solving civil engineering problems can be very large scale and can be highly nonlinear, and to find solutions to these problems is often very challenging. In the past two decades, soft computing methods are becoming an important class of efficient tools for developing intelligent systems and providing solutions to complicated engineering problems.

The papers selected for this special issue represent a good panel in recent challenges. The topics of these papers are connected with the computational intelligence methods and their application in civil and hydraulic engineering. An investigation on different metaheuristics abilities for engineering problems was studied by O. Adekanmbi and P. Green. They utilized the third version of generalized differential evolution (GDE) for solving practical engineering problems. GDE3 metaheuristic modifies the selection process of the basic differential evolution and extends DE/rand/1/bin strategy.

Performance-based seismic design of steel frames using the colliding bodies optimization (CBO) algorithm as new optimization method was presented by H. Veladi. A pushover analysis method based on semirigid connection concept was developed and the CBO algorithm is employed to find optimum seismic design of frame structures. H. Veladi solved two numerical examples from literature and show the power or weakness of this new algorithm.

A parametric study of nonlinear seismic response analysis of transmission line structures and the effect analysis of strain rate on power transmission tower-line system under seismic excitation were developed by L. Tian et al. in two papers. In one of them, nonuniform ground motions are generated using a stochastic approach based on random vibration analysis. Then, the effects of multicomponent ground motions, correlations among multicomponent ground motions, wave travel, coherency loss, and local site on the responses of the cables were investigated using nonlinear time history analysis method. The results showed the multicomponent seismic excitations should be considered, but the correlations among multicomponent ground motions could be neglected. While, in the other, a three-dimensional finite element model of a transmission tower-line system was created based on a real project. The results showed that the effect of strain rate on the transmission tower generally decreases the maximum top displacements, but it would increase the maximum base shear forces, and thus it is necessary to consider the effect of strain rate on the seismic analysis of the transmission tower. The effect of strain rate could be ignored for the seismic analysis of the conductors and ground lines, but the responses of the ground lines considering strain rate effect are larger than those of the conductors.

A study of bridge structures is performed by N. Easazadeh Far and M. Barghian. They selected integral abutment bridges (IABs) as jointless bridges. Although all developed bridge design codes consider temperature and earthquake loads separately in their specified load combinations for conventional bridges with expansion joints, the thermal load is an “always on” load and during the occurrence of an earthquake, these two important loads act on bridge simultaneously. Safety identifying of these bridges under seismic and thermal loads is the main aim of their work where the safety of IABs, designed by AASHTO LRFD bridge design code, under combination of thermal and seismic loads was studied. They showed that for an IAB designed by AASHTO LRFD the reliability indexes have been reduced under combined effects.

In the field of hydraulic engineering, optimal design of pipe sizes for looped irrigation water supply system is presented by D. G. Yoo et al. in which they developed a harmony search algorithm to fulfill this aim. Their study mainly serves two purposes. The first is to develop an algorithm and a program for estimating a cost-effective pipe diameter for agricultural irrigation water supply systems using optimization techniques. The second is to validate the developed program by applying the proposed optimized cost-effective pipe diameter to an actual study region (Saemangeum project area, zone 6). They show that the optimal design program can be effectively applied for the real systems of a looped agricultural irrigation water supply.

